# Attachment style of patients diagnosed with psychogenic non-epileptic seizures at a tertiary Epilepsy Center in the Czech Republic

**DOI:** 10.3389/fpsyt.2022.1065201

**Published:** 2022-11-17

**Authors:** Lenka Krámská, Lucia Hrešková, David Krámský, Zdeněk Vojtěch

**Affiliations:** ^1^Department of Clinical Psychology, Na Homolce Hospital, Prague, Czechia; ^2^Epilepsy Center, Na Homolce Hospital, Prague, Czechia; ^3^Kúpele pre dušu, s.r.o., Bardejov Spa, Bardejov, Slovakia; ^4^Department of Social Science, Police Academy, Prague, Czechia

**Keywords:** psychogenic non-epileptic seizures, attachment, family relationships, childhood stressors, functional neurological disorders

## Abstract

**Objective:**

Quality and type of early relationships with primary caregivers is considered one of the key factors in the etiopathogenesis of many mental disorders including depression, anxiety, and conversion disorders. This study focused on the type and quality of attachment style in adult patients with psychogenic non-epileptic seizures (PNES).

**Materials and methods:**

We evaluated the demographic data and profiles of PNES patients (*n* = 262) and group of healthy volunteers (*n* = 51) measured by the Parental Bonding Inventory (PBI) and Experiences in Close Relationships (ECR) and Experiences in Close Relationships–Relationship Structure (ECR-RS).

**Results:**

Significant differences in measured values between the two groups were identified; specifically, differences in the caregiver style–father and mother overprotection (PBI) was higher in the PNES group. The most frequent type of attachment in PNES was type 2 (preoccupied). Correlations between the PBI and ECR results were also found.

**Conclusion:**

This study highlighted certain attachment styles in patients with PNES and statistically significant differences between patients with PNES and a healthy sample. Some correlations between the results of the questionnaires with socio-demographic factors were found. The identification of specific patterns in attachment may be useful for further use in reaching a differential diagnosis and administering tailored psychotherapy of patients with PNES.

## Introduction

Psychogenic non-epileptic seizures (PNES) are sudden and involuntary episodic events (paroxysmal), manifested by motor, sensory, and mental or autonomic symptoms that resemble epileptic seizures (ES). In PNES, normal functioning of the central nervous system is altered, and self-control is weakened (1), but these symptoms are not caused by epileptogenic activity (2).

In addition to the more traditional research on psychological and environmental factors of PNES, the significance and meaning of attachment in the development of PNES has also been studied in last years.

According to Bowlby’s attachment theory, adaptation to new experiences is strongly determined by the emotional relationships formed during childhood. Different types of attachment influence the creation of multiple mental representations of self and others (3, 4). Bowlby described the separation reactions of hospitalized children aged 18–24 months, dividing these reactions into the following stages–protest, despair and detachment from the mother. He laid the foundation for the various emotional attachment types that were further developed by Mary Ainsworth and Margaret Mahler. Secure attachment–children show distress when separated and joy when reunited. Children may be upset, they feel assured that the caregiver will return. Avoidant type–Children tend to avoid caregivers, they do not show signs of dissatisfaction when separated from them. It might be a result of neglectful or abusive caregiver. Children who are punished for relying on a caregiver will learn to avoid seeking care in the future and suppress emotions. Disorganized type–It is linked to inconsistent caregiver behavior. Parents may serve as both a source of fear and safety (typical for parents suffering from psychosis or substance abuse). These children produce a confusing mix of behavior without any logical pattern, which is disoriented, dazed, or confused. Ambivalent type–children become very anxious, and they react very affectively when a caregiver leaves. They are unable to interact with a stranger without caregiver, and when caregiver returns, they are dependent on caregiver, or react angrily and try to resist his attempts to approach them. They are afraid of being abandoned again by the caregiver.

The predominant attachment type shapes individual behavioral patterns for how they should behave in future relationships (provides information about future interpersonal behavior). Attachment also has an important role in the formation of emotional regulation skills. Dysfunctional attachment in childhood is often associated with maladaptive interpersonal interactions and functioning, that increases vulnerability to adult psychopathology disorders (4, 5).

A possible explanatory association between high rates of dissociation and psychopathological conditions and high rates of childhood trauma could be clarified by the attachment theory (6).

Seizure disorders are paroxysmal and unpredictable events, resulting in highly variable levels of care need (7). Seizures do not only affect the patients, but also their families, relationships with partners and friends (8). This can arise through the assumption of a caring role in which the caregiver makes vital contributions to the management of chronic illnesses. However, there is also an impact on caregivers’ wellbeing, and many caregivers report that their mental health has worsened as a result of caregiving.

In the study of Wardrope (7) the trait most strongly associated with caregivers wellbeing in patients with PNES (CfPNES) was the presence of an anxious attachment style in caregivers.

In addition, the patient’s individual attachment behavior might be essential for the outcome of therapy and should be taken into account in the therapeutic process (9, 10).

In our study, we aimed to explore the role of different attachment styles, measured by a questionnaire Experience in Close Relationships (ECR), and Experience in Close Relationships–Relationship Structure (ECR-RS), and Parental Bonding Instrument (PBI) in large sample of patients diagnosed with PNES, by further comparing our PNES sample to a smaller group of healthy volunteers.

The basic hypothesis was that pathological attachment styles will be increased in patients with PNES and be more prevalent among patients with PNES than in the group of healthy volunteers. A secondary hypothesis is that there exists relationship between attachment styles measured by PBI and ECR questionnaires.

## Materials and methods

### Study design and target population

In this retrospective study comprehensive neuropsychological evaluation was performed on 262 patients [*N* = 262; F:M 191:71; mean age 38.4 (13.4)] diagnosed with PNES through v- EEG monitoring at the Na Homolce Hospital Epilepsy Center in Prague, Czech Republic during the period of 2010–2021. Healthy volunteers [*N* = 51; F:M 37:14; mean age 28.6 (8.2)] underwent same test battery from 2018 to 2019. Data collection was carried out by random sampling, mainly among university students (graduate, post-graduate or continuing education).

#### Neurological examination

Patients were admitted to an epilepsy monitoring unit at Na Homolce Hospital, Prague, the Czech Republic. There was obtained a detailed medical history and neurological examination by a certified epileptologist (ZV) was performed. Patients underwent long-term video-EEG monitoring, routine laboratory tests, and high-resolution magnetic resonance imaging. The length of stay was 7 days. If the patient didn’t have a seizure, suggestive induction of habitual seizure was performed at the end of their stay (7 days). Inclusion criteria were: (a) age equal to or more than 18 years old; (b) a VEEG confirmed diagnosis of PNES only.

#### Neuropsychological examination

Patients with PNES were examined by a licensed clinical neuropsychologist (LK) who acquired a detailed personal history (family, medical, psychiatric, occupational, stressors and traumatic events, etc.), and administered a cognitive battery, personality assessment, and mood status evaluation. The ECR, ECR-RS, and PBI were used to describe an attachment style. Czech ECR questionnaires were added to neuropsychological battery in 2019 (explaining the smaller number of participants). A comprehensive evaluation was performed during 2 days, each lasting 2–3 h on average. Patients signed an informed consent at admission to the Epilepsy Center. They were informed about assessment results at the end of their hospitalization. Healthy volunteers underwent same test battery. The inclusion criteria were the absence of: neurological, psychiatric, and somatic diseases, substance abuse, pharmacotherapy influencing cognitive performance in personal history; psychiatric disorder in family history. Data collection was carried out by random sampling, mainly among university students (graduate, post-graduate or continuing education).

### Psychometric measures

#### Experiences in close relationships (ECR)

This is a 36-item measure of adult attachment style that measures individuals on two subscales of attachment: Avoidance and Anxiety. In general Avoidant individuals find discomfort with intimacy and seek independence, whereas Anxious individuals tend to fear rejection and abandonment (11). The measure used is a Czech translation made in 2012 with preservation of corresponding psychometric characteristics (11, 12). To determine a specific type of bond, the authors (13) developed a calculation and determination of a specific type of relational bond–SEC stands for secure dimension (secure attachment), PRE denotes the preoccupied dimension (preoccupied bond type), DIS stands for dismissing dimension (distant type of bond), and FEAR indicates the fearful dimension (fearful attachment). Study that map the psychometric properties of the questionnaire (14). The ECR and ECR-RS questionnaires have adequate criterion and construct validity, as well as reliability in terms of internal consistency of the scales. Internal consistency using Cronbach’s α for both scales, when the relational anxiety scale reached a value of 0.84 and the relational avoidance scale also reached 0.84.

#### Experiences in close relationships–relationship structure (ECR–RS)

The Relationship Structure Questionnaire measures global as well as specific emotional attachment to specific close people (mother, father, partner, and best friend). Like the ECR, the ECR-RS is based on the measurement of two dimensions related to attachment patterns, avoidance and anxiety (11, 15). The ECR-RS measures relational avoidance and anxiety scores for each specific attachment (mother, father, partner, and best friend). In relation to each person mentioned above, the respondent evaluates nine statements on a seven-point scale from “I strongly disagree” to “I strongly agree.” It evaluates a total of 36 statements. Psychometric characteristics of the questionnaire is described in the study of Rocha et al. (16). Cronbach’s alpha reaches similar values to ECR (the lowest measured value was 0.85, the highest 0.92, and for ECR and ECR-R the values usually reach around 0.91). The reliability of global anxiety and global avoidance calculated by averaging the scores obtained on the ECR-RS for individuals was also satisfactory (0.85 for anxiety and 0.88 for avoidance at *p* < 0.05).

#### Parental bonding instrument (PBI)

Two scales termed “care” and “overprotection” or “control,” measure essential parental styles as perceived by the child. It is “retrospective” measure, meaning that adults (over 16 years) complete the evaluation for how they remember their parents during their first 16 years. The measure is to be performed for both mothers and fathers separately. There are 25 item questions, including 12 “care” items and 13 “overprotection” items (17). Based on the two parenting dimensions, following types of parenting style were identified: average; high care and low overprotection conceptualized as optimal parenting; low care and high overprotection conceptualized as affectionless control; low care and low overprotection conceptualized as neglectful parenting; and, high care and high overprotection conceptualized as affectionate constraint.

The cut-off scores for the study from 1979 and for the Czech study do not differ, the cut-off score was 25 for the mother’s care scale, 21 for the father’s, 15 for the mother’s control scale, and 13 for the father’s (18).

### Data analysis

The data was compiled and analyzed by frequency and percentage tables using statistical software Statistica vX. A.

The statistical analysis of the data was based on the research hypotheses, which represents.

(1) Verification of the difference in the distribution of the scales of both questionnaires (PBI and ECR/ECR-RS) between the diagnostic and control groups; (2) Analysis of statistical dependence between the scales of both questionnaires (PBI and ECR/ECR-RS).

For the analysis, we chose conservative non-parametric methods. Differences in the distribution of the scales between the two groups are tested using the two-sample Wilcoxon test (in addition to the basic statistics, we present the difference in mean, median, value of the respective test z-statistic together with the *p*-value in the tables). The difference of the distribution of the categorical variable “bond type” is tested by the classical Chi-square Goodness of Fit Test.

We analyzed the dependence between the scales of the questionnaires using the Spearman correlation coefficient (corrected for continuity). Statistical analysis of the data was performed at a significance level of α = 0.05 (95%).

### Ethics

All data collection, analysis, storage, and processing were done in compliance with the Helsinki Declaration. All patients signed informed consent and the study was approved by the Ethical Committee of the Na Homolce Hospital.

## Results

### Study 1: Comparison of parental bonding instrument between psychogenic non-epileptic seizures patients and healthy volunteers

In the first portion of the study the attachment style in both patients with PNES and healthy volunteers including their demographic characteristics were examined. With regard to gender, the two groups were balanced. The mean age was higher in patients’ group. The length of schooling was higher in the group of healthy volunteers (Table 1).

**TABLE 1:** Demographic variables of patients and healthy volunteers for Parental Bonding Inventory (PBI).

	Patients	Healthy volunteers		
Gender	191 (72.9%) w	37 (72.5%) w	Pearson χ^2^	*p* = 0.983
	71 (27.1%) m	14 (27.5%) m		
Age	38.4 (13.4)	28.6 (8.2)	Z (MANN-WH) = 5.05	*P* < 0.001
Education (years)	12.3 (2.1)	15.4 (1.8)	Z (MANN-WH) = −8.98	*P* < 0.001

w, women; m, men.

When we compared both groups, we found statistically significant differences in the caregiver styles in 50% of subscales–father and mother overprotection was higher in the PNES group. Overprotection scale was also slightly higher than cut-off scores in both mother (15 points) and father (13 points). In the PNES group we also detected slightly lower mothers care in comparison to the cut-off score (25 points). The difference in mothers care between both groups was 6.2 points (with higher care in control group), but was not statistically significant (Table 2).

**TABLE 2:** Comparison of the caregiver style between patients and volunteers.

PBI	Patients *n* = 262			Controls *n* = 51					Wilcoxon test	
			
	Mean	Median	SD	Mean	Median	SD	Mean difference	Median difference	Z	*P*
Care F	20.8	22.0	11.2	21.6	22.0	9.5	–0.8	0.0	−0.23	0.818
Overprotection F	13.5	12.0	7.5	10.5	8.0	6.6	3.0	4.0	−2.87	0.004
Care M	23.8	27.0	11.3	27.5	30.0	7.7	–3.7	–3.0	−1.54	0.122
Overprotection M	15.1	15.0	7.6	12.1	11.0	6.9	3.0	4.0	−2.62	0.009

F, father; M, mother.

### Study 2: Comparison of experiences in close relationships and experiences in close relationships–relationship structure between psychogenic non-epileptic seizures patients and healthy volunteers

In the portion of the second study, the attachment styles measured by ECR in both patients with PNES and healthy volunteers including their demographic characteristics were examined. There were no differences in gender between both groups. The length of schooling was higher in the group of healthy volunteers. The mean age was higher in group of patients (Table 3).

**TABLE 3:** Demographic variables of patients and healthy volunteers for Experiences in Close Relationships (ECR) and Experiences in Close Relationships–Relationship Structure (ECR-RS).

	Patients	Healthy volunteers		
Gender	40 (70.2%) w	37 (72.5%) w	Pearson χ^2^	*p* = 0.785
	17 (29.8%) m	14 (27.5%) m		
Age	36.1 (12.3)	28.6 (8.2)	Z (MANN-WH) = 3,33	*P* < 0.001
Education (years)	12.8 (2.4)	15.4 (1.8)	Z (MANN-WH) = −6,17	*P* < 0.001

w, women; m, men.

In PNES group of patients, the most frequently detected type of attachment overall was type 2 (preoccupied dimension) (*n* = 27, 47.4%). The least frequent type 1 (secure dimension) (*n* = 7, 12.3%) and type 3 (dismissing dimension) (*n* = 7, 12.3%). In the control group, type 4 (fearful dimension) was the most common (*n* = 19, 37.3%). A Chi-Square Goodness of Fit Test was performed to determine whether the proportion of attachment type was equal between two groups. The proportions did not differ by attachment type X^2^ (3, *N* = 77) = 4.656, *p* = 0.199. Detailed results are in Table 4.

**TABLE 4:** Comparison of the attachment type between patients and healthy volunteers.

Attachment type		Patients	Control
(1) Secure dimension	FR	7	10
	%	12.3%	19.6%
(2) Preoccupied dimension	FR	27	14
	%	47.4%	27.5%
(3) Dismissing dimension	FR	7	8
	%	12.3%	15.7%
(4) Fearful dimension	FR	16	19
	%	28.1%	37.3%
	FR	57	51
	%	100.0%	100.0%
**Pearson Chi-Square**	**Value**	**df**	***P***
	4.656	3	0.199

A statistically significant differences between both groups were detected in 50% of measured variables: ECR Avoidance, ECR-RS Anxiety mother, ECR-RS Anxiety father, ECR-RS Avoidance partner, ECR-RS Avoidance best friend (for all measures *p* < 0.05). These scales were increased in PNES patients in comparison to control group. Detailed results are in Table 5.

**TABLE 5:** Comparison of the attachment structure between patients and healthy volunteers.

ECR and ECR-RS	Patients *N* = 58			Controls *N* = 51						
			
	Mean	Median	SD	Mean	Median	SD	Mean difference	Median difference	Wilcoxon test	
									
									Z	*p*
ECR avoidance	3.90	3.97	0.98	3.30	3.33	0.81	0.60	0.64	−3.37	0.001
ECR anxiety	3.64	3.61	0.96	3.50	3.22	1.00	0.14	0.39	−0.97	0.333
ERC RS avoidance M Mean	3.73	3.42	1.66	3.31	3.17	1.33	0.42	0.25	−1.14	0.254
ECR RS anxiety M Mean	2.46	1.83	1.69	1.56	1.00	1.05	0.90	0.83	−2.88	0.004
ERC RS avoidance F Mean	4.03	4.00	1.35	4.10	4.00	1.39	–0.07	0.00	−0.04	0.968
ECR RS anxiety F Mean	2.44	2.00	1.68	1.58	1.00	0.93	0.86	1.00	−2.75	0.006
ERC RS avoidance P Mean	3.10	3.17	1.48	2.27	2.00	1.06	0.82	1.17	−2.88	0.004
ECR RS anxiety P Mean	2.89	2.33	1.99	2.69	2.00	1.65	0.20	0.33	−0.24	0.808
ERC RS avoidance BF Mean	3.42	3.50	1.48	2.49	2.50	0.99	0.93	1.00	−3.59	<0.001
ECR RS anxiety BF Mean	2.78	2.00	1.98	1.94	1.33	1.34	0.84	0.67	−1.92	0.055

M, mother; F, father; P, partner; BF, best friend.

A statistically significant negative correlations were found between the PBI–Father care and ECR-RS–Father Avoidance [rs (54) = −0.59, *p* < 0.001]; PBI–Mother care and ECR–Avoidance [rs (57) = −0.34, *p* = 0.010]; PBI–Mother care and ECR-RS–Mother Avoidance [rs (57) = −0.56, *p* < 0.001]; PBI Mother care and ECR-RS–Father Avoidance [rs (56) = −0.47, *p* < 0.001]. A statistically significant positive correlation was found between the PBI–Father overprotection and ECR-RS Father avoidance [rs (54) = 0.32, *p* < 0.018] and PBI–Mother overprotection and ECR-RS Mother avoidance [rs (58) = 0.42, *p* < 0.001]. Detailed results are shown in the Table 6.

**TABLE 6:** Spearman correlation between Parental Bonding Inventory (PBI) and Experiences in Close Relationships (ECR) in patients’ group.

PBI		ECR avoidance	ECR anxiety	ERC-RS avoidance M mean	ECR-RS anxiety M mean	ERC-RS avoidance F mean	ECR-RS anxiety F mean	ERC-RS avoidance P mean	ECR-RS anxiety P mean	ERC-RS avoidance BF mean	ECR-RS anxiety BF mean	ERC-RS avoidance OL mean	ECR-RS anxiety OL mean
Care F	R	0.02	−0.06	−0.24	−0.20	−0.59	−0.16	0,12	0.01	−0.20	−0.10	−0.06	0.01
	P	0.910	0.663	0.077	0.136	<0.001	0.242	0.386	0.962	0.143	0.479	0.655	0.963
	N	55	55	55	55	54	54	53	53	54	54	55	55
Overprotection F	R	0.09	−0.02	0.19	0.08	0.32	0.24	0.01	0.05	0.01	0.16	−0.01	−0.25
	P	0.524	0.880	0.156	0.544	0.018	0.079	0.952	0.696	0.954	0.255	0.970	0.060
	N	55	55	55	55	54	54	53	53	54	54	55	55
Care M	R	−0.34	−0.04	−0.56	−0.13	−0.47	−0.11	−0.06	0.11	−0.19	−0.14	−0.06	−0.09
	P	0.010	0.756	<0.001	0.351	<0.001	0.424	0.654	0.421	0.162	0.305	0.663	0.529
	N	57	57	57	57	56	56	56	56	56	56	57	57
Overprotection M	R	0.12	0.12	0.42	−0.06	0.16	0.02	−0.24	0.02	−0.03	−0.01	0.25	−0.24
	P	0.365	0.377	0.001	0.656	0.241	0.872	0.079	0.880	0.821	0.921	0.059	0.067
	N	58	58	58	58	57	57	56	56	57	57	58	58

M, mother; F, father; P, partner; BF, best friend.

## Discussion

Measuring emotional attachment is not a common and widespread clinical practice and there are even fewer studies that examine the relationship between PNES and attachment, or family relationships in general. However, there are some studies that demonstrate interest in this issue (7, 19, 20).

Focusing on the investigation of attachment could serve as one of the important elements of clinical-psychological differential diagnosis (in addition to anamnesis and compliance, diagnosis of cognitive abilities, motor and executive functions, personality traits, psychopathology, etc.) (21).

In the current work, we aimed to explore the role of different attachment styles, defined by a questionnaire ECR and PBI and its relationship with PNES, by further comparing our PNES sample to a small group of healthy volunteers.

The results of our study point to the existence of certain attachment styles in patients with PNES and statistically significant differences between patients with PNES and the control group.

When we compared both groups, we found statistically significant differences in the caregiver styles–father and mother overprotection was higher in the PNES group. The Overprotection scale was also slightly higher than recommended cut-off scores. In the PNES group was found slightly lower mothers care in comparison to the cut-off score. We also detected differences in mothers’ care between both groups with higher care in healthy volunteers, but it did not reach statistical significance.

Lahousen et al. (22) explain the emergence of emotional attachment disorders in childhood as a consequence of traumatic experiences in relationships with important attachment figures. These experiences can be caused by, for example, a lack of sensitivity and care for the child’s needs on the part of the caregiver, abuse, etc. Ludwig et al. (23) in their large study concluded that stressful life events and maltreatment are substantially more common in people with the functional neurological disorder than in healthy controls and patient controls. Emotional neglect had a higher risk than traditionally emphasized sexual and physical abuse, but many cases report no stressors. These disorders then contribute to the creation of a so-called vicious circle, in which the individual is unable to regulate his emotions, causes negative reactions in those around him with this behavior and, as a result, through the rejecting reactions of the environment, it confirms the original schemas and beliefs about the world as a hostile, dangerous place.

The most frequent attachment style in the PNES group was type 2 (preoccupied dimension) and the least frequent type 1 (secure dimension). The Preoccupied type of attachment is thought to be a psychological defense against the devastating effects of trauma, or somatic symptoms resulting from repressed intrapsychic conflicts leading to dissociation, which is the most common defense mechanism in these patients (24).

The form of an emotional bond in childhood is substantial as a result of influences resulting in adulthood, including awareness of one’s value, and the creation of models according to which we establish intimate interpersonal relationships in adulthood. Insecure and fearful attachment is also related to perceived distrust of other people and resilience (25). In the control group, type 4 (fearful dimension) was the most common. The possible explanation is, that the control group consisted primarily of university students and younger adults. In general, this period of life is associated with opening up to new relationships and therefore with more frequent disappointments and resulting fears (26). They were also in a weaker position in their role of student vis a vis professors, being examined and graded. Another explanation is that many students and young adults are also in a developmental period when they may not have worked out the topic of relationships with authorities, including parents. Although the students have reached adulthood, they are still economically and materially dependent on their parents.

Regarding attachment style and structure, statistically significant differences between both groups were detected in the following variables, with higher scores in the PNES group: ECR Avoidance, ECR-RS Anxiety mother, ECR-RS Anxiety father, ECR-RS Avoidance partner, and ECR-RS Avoidance best friend. These results could correspond to predisposing factors for the development of PNES, such as a traumatic events in personal history, situational triggers, and specific personality traits, and emotional suppression and emotional overwhelming (24, 27, 28).

We also compared results of both methods we used in our study. Statistically significant negative correlations were found between the PBI—Father care and ECR-RS–Father Avoidance; PBI–Mother care and ECR–Avoidance; PBI–Mother care and ECR-RS–Mother Avoidance; PBI Mother care and ECR-RS–Father Avoidance. In sum, this would suggest that the less parental care occurs, the higher level of avoidance behavior is associated with it.

A positive correlation was found between the PBI–Father overprotection and ECR-RS Father avoidance and PBI–Mother overprotection and ECR-RS Mother avoidance. We can conclude that a high degree of control is also associated with a higher degree of emotional avoidance. Patients with PNES were found to have lower levels of caregiving and higher levels of control from parents; controlling parents tend to be less emotionally available and avoid emotional closeness. Subsequently, this tends to increase anxiety in children and leads to suppression of their own emotions.

Dysfunctional communication in the family system is often mentioned in connection with families of PNES patients (23, 29). Among the consequences of dysfunctional communication, the authors describe more frequent conflicts and a lack of emotional acceptance, all of which are perceived primarily by patients with PNES, and not by other family members. The question remains whether anxiety in relation to parents is related to the patient’s diagnosis, or whether it preceded that diagnosis (29).

These findings suggest that is may be important to pay attention to test methods that determine the nature of the emotional bond as part of the differential diagnosis of PNES, but also, for example, as tools to find out information about the patient’s family environment that can subsequently be useful in psychotherapy (30).

Attachment theory provides an association between early traumatic events, family dysfunction, and psychopathological conditions. According to this theory, early childhood interactions with primary caregivers shape patterns of beliefs, thoughts, emotions, and behaviors regarding others and self, conceptualized as attachment styles. Attachment disorders have been linked to several mental disorders including PNES. Compared to the control group, adolescents with PNES had more emotional and traumatic sexual experiences and PTSD symptoms. Patients with PNES perceived higher “communication” but lower “trust” in attachment relationships with their mothers and fathers (31).

The predominance of insecure and fearful attachment was described in patients with PNES (10). Insecure and disorganized attachment types have previously been associated with psychiatric disorders, including functional neurological disorders (10, 32, 33). Patients with PNES use less mature defensive strategies, which again might be linked to insecure attachment patterns (4, 10). Dysfunctional family relationships and insecure attachment styles have been detected in patients with PNES but also patients with epilepsy (34).

It has been also argued that preoccupied attachment style might complicate the engagement in the therapeutic treatment and the alliance with the therapist (35).

It would be appropriate to investigate attachment with significant others more in detail, for example, the relationship between upbringing and approach in the client’s primary family (control and care subscales in the PBI questionnaire) and between attachment types. Another topic that could be explored in more detail is the relationship between global and specific emotional attachment in patients with PNES. It is also important to focus on the relationship and connections between types of emotional attachment, occurrence of traumatic events in childhood and specific coping mechanisms.

There are some limitations of this study. The research and control group are inhomogeneous in some socio-demographic characteristics (age and education). It was mainly due to the limited possibilities of data collection during the COVID pandemic, and due to the willingness of healthy volunteers to participate in the study. Another limitation is that we didn’t perform the multivariate analysis due to the characteristic of both samples and the inclusion of two groups only. Due to the low number of participations represented in the research sample, this work has limits in terms of generalization and transferability of the results to the PNES population, as part of possible further studies, it would be preferable to increase the research sample.

Due to the high values of anxiety or avoidance found in some subscales in the group of patients with PNES and comorbidities related to this disease, further research could possibly be supplemented with scales measuring subjectively perceived anxiety. Also, questionnaires mapping character of the patient’s interpersonal interactions, all ideally provided that the patient is measured before and after completing psychotherapy, would be recommended. Such research could help describe the effects of different types of psychotherapy in patients with PNES.

According to the available literature, there is empirical evidence of the effect of these therapeutic approaches: cognitive behavioral therapy (36), long-term exposure therapy (37), psychodynamic therapy (38), focused therapy on mindfulness (39), and group dialectical behavior therapy (21). New findings in the psychotherapy of patients with a diagnosis of PNES [for example, (37)] emphasize the importance of focus to therapeutic work with traumatic events in the anamnesis.

## Conclusion

The results of our study point to the predominance of certain attachment styles in patients with PNES and statistically significant differences between patients with PNES and a group of healthy volunteers. Our research has shown that there are possible connections of the results of used questionnaires with socio-demographic and psychosocial factors that could be the subject of further investigation. Attachment type can be a valuable and important psychological factor for differential diagnosis, but above all for a deeper understanding of the etiopathogenetic factors involved in the genesis of PNES in a specific patient. This can be especially useful in deciding on a treatment plan or approach for individual patients.

## Data availability statement

The original contributions presented in the study are included in the article/supplementary material, further inquiries can be directed to the corresponding author.

## Ethics statement

The studies involving human participants were reviewed and approved by the Ethical Committee of the Na Homolce Hospital. The patients/participants provided their written informed consent to participate in this study.

## Author contributions

LK prepared the conception and design of the study and participated in the acquisition and analysis of data. LK and LH wrote the original draft of the manuscript and critically revised the manuscript. ZV and DK helped in the analysis of data and critically revised and edited the manuscript. All the authors met the standard criteria of authorship based on recommendations of the International Committee of Medical Journal Editors.
